# Láser de baja potencia como coadyuvante a la parestesia. reporte de dos casos

**DOI:** 10.21142/2523-2754-1202-2024-201

**Published:** 2024-06-27

**Authors:** Alejandra Brítez, Luján Martínez, Virginia Sanabria, María del Carmen González, Fátima Torres Marín, José Miguel Gamarra, Gloria Galeano

**Affiliations:** 1 Facultad de Odontología Universidad Nacional de Asunción. Asunción, Paraguay. alejandrabritez@founa.edu.py , lujanmartinez@founa.edu.py , virginiasanabria@founa.edu.py , maricarmen.ggalva@gmail.com , fatitorresmarin@gmail.com , josemgamarra31@gmail.com , gloriagaleano@odo.una.py Universidad Nacional de Asunción Facultad de Odontología Universidad Nacional de Asunción Asunción Paraguay alejandrabritez@founa.edu.py lujanmartinez@founa.edu.py virginiasanabria@founa.edu.py maricarmen.ggalva@gmail.com fatitorresmarin@gmail.com josemgamarra31@gmail.com gloriagaleano@odo.una.py

**Keywords:** terapia por láser, parestesia, cirugía bucal, laser therapy, paresthesia, surgery oral

## Abstract

La extracción dentaria o exodoncia es un procedimiento común en la práctica odontológica, si bien no está exento de presentar complicaciones. La parestesia es definida como una neuropatía con alteración de sensaciones y anestesia permanente. Se reporta el caso de dos pacientes con parestesia que acudieron a la cátedra de patología de la Facultad de Odontología. Ambos fueron tratados con terapia láser de baja potencia, lo que mostró un mejoramiento significativo en su cuadro clínico. La aplicación del láser de baja potencia presenta beneficios idóneos en diversas áreas del campo estomatológico. Al ser un tratamiento no invasivo, indoloro y con sesiones de corta duración, estimula al paciente a continuar con el tratamiento hasta estar casi totalmente rehabilitado.

## Abstract (en)

Tooth extraction or extraction is a common procedure in dental practice, although it is a common practice, it is not exempt from complications. Paraesthesia is defined as a neuropathy with altered sensations and permanent anesthesia. The case of two patients who attended the Department of Pathology at the Faculty of Dentistry with paresthesia is reported. Both were treated with low-power laser therapy, showing a significant improvement in their clinical condition. The application of low-power laser presents ideal benefits in various areas of the stomatological field. As it is a non-invasive, painless treatment and short sessions, the patient is encouraged to continue with the treatment until they are almost completely rehabilitated.

## INTRODUCCIÓN

La extracción dentaria o exodoncia es un procedimiento común en la práctica odontológica, si bien no está exento de presentar complicaciones. Las complicaciones pueden ser clasificadas como intraoperatorias y posoperatorias. Las posoperatorias se clasifican en infecciones, abscesos y fascitis necrotizante; y las no infecciosas, en dolor, hemorragia, edema, alveolitis, parestesia, comunicación sinusal, trismo, enfisema y desórdenes temporomandibulares ^1^^-^^4^.

La parestesia es definida como una neuropatía con alteración de sensaciones y anestesia permanente. En la odontología, las parestesias faciales se manifiestan frecuentemente por medio de los nervios alveolar inferior, lingual, mentoniano, trigémino y facial, y son producto de factores locales o sistémicos ^5^.

Las parestesias tienen diversas causas, entre las cuales se podría mencionar las mecánicas (traumas físicos de los nervios como compresión, estiramiento o ruptura), las patológicas (tumores cuyo crecimiento provoca compresión del nervio en la región y daña las fibras nerviosas sensitivas), las físicas (exceso de calor, como en el caso de realización de osteotomía con instrumentos rotatorios sin una adecuada refrigeración), las químicas (aplicación de anestésicos locales o sustancias en las inmediaciones del nervio) y las microbiológicas (infecciones en tejidos blandos y duros) ^6^.

Entre las técnicas actuales para aliviar la sintomatología del paciente con parestesia, la literatura señala algunas posibles acciones del láser de baja potencia, como la regeneración acelerada del tejido nervioso lesionado y la estimulación de los tejidos nerviosos adyacentes para que desempeñen el papel del tejido nervioso lesionado ^7^. En este sentido, este reporte de dos casos buscó evidenciar el beneficio de la aplicación del láser de baja potencia en pacientes con parestesia.

## REPORTE DE CASOS

### Primer caso

Un paciente de sexo masculino, de 29 años, acude a la clínica de patología bucal de la Facultad de Odontología de la Universidad Nacional de Asunción. Este refiere adormecimiento y pérdida de la sensibilidad en la zona hemifacial del lado derecho, así como dolor en la zona del músculo esternocleidomastoideo del mismo lado. 

Durante la anamnesis, comentó que la aparición de los síntomas coincidió con unas exodoncias de las piezas 1.5 (segundo premolar superior derecho) y 4.8 (tercer molar inferior derecho), con un tiempo de evolución de tres meses, lo cual repercutió mucho en su desempeño psicosocial y personal.

**Figura 1:**
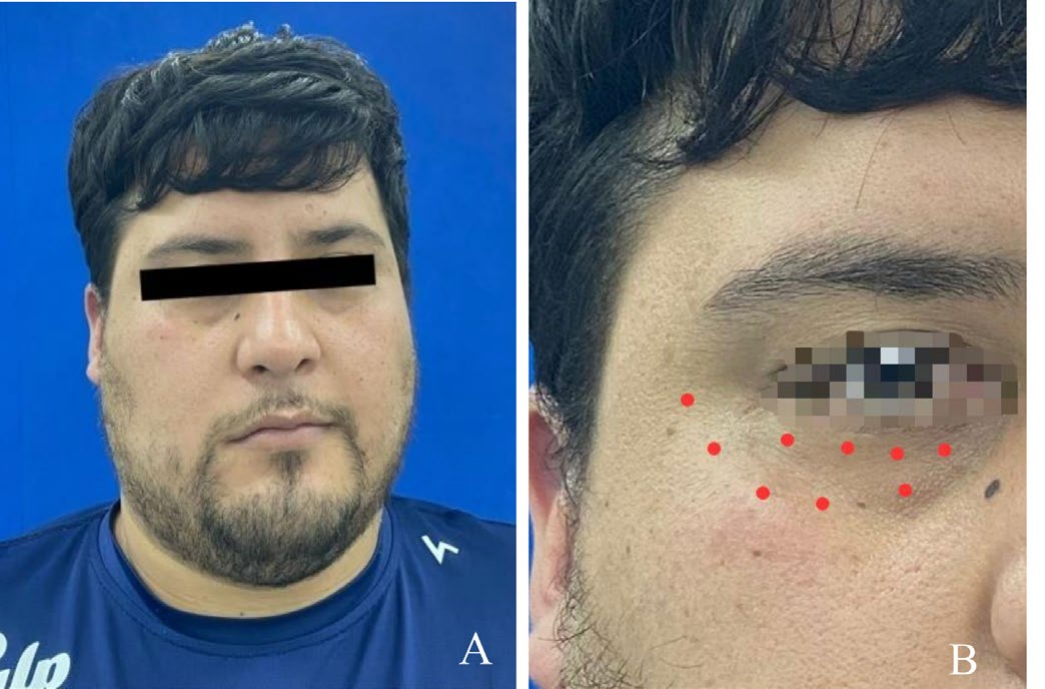
Fotografías extraorales. A) Fotografía de frente donde se evidencia falta del tono muscular del lado derecho. B) Zona de afectación: orbicular y palpebral inferior.

En la exploración clínica extraoral, se evidenció la pérdida del tono muscular de toda la zona facial derecha, la falta de sensibilidad a nivel orbicular y palpebral inferior, y una baja función motora, lo que dificulta al paciente el cierre ocular, con esfuerzo y en reposo, así como sonreír, realizar gestos y parpadear, lo que ocasiona sequedad ocular (Figuras 1a y 1b).

Teniendo en cuenta la anamnesis y la exploración clínica, se estableció como diagnóstico una parestesia hemifacial del lado derecho, relacionada con la intervención quirúrgica a la que fue sometido. La parestesia es una de las complicaciones más comunes de este tipo de procedimientos. El diagnóstico fue realizado por la especialista en medicina oral y patología bucal. Como tratamiento, se indicaron 5 sesiones de láser de baja potencia, 15 sesiones de fisioterapia de electroestimulación (5 veces a la semana) y el uso de lágrimas artificiales. 

**Figura 2:**
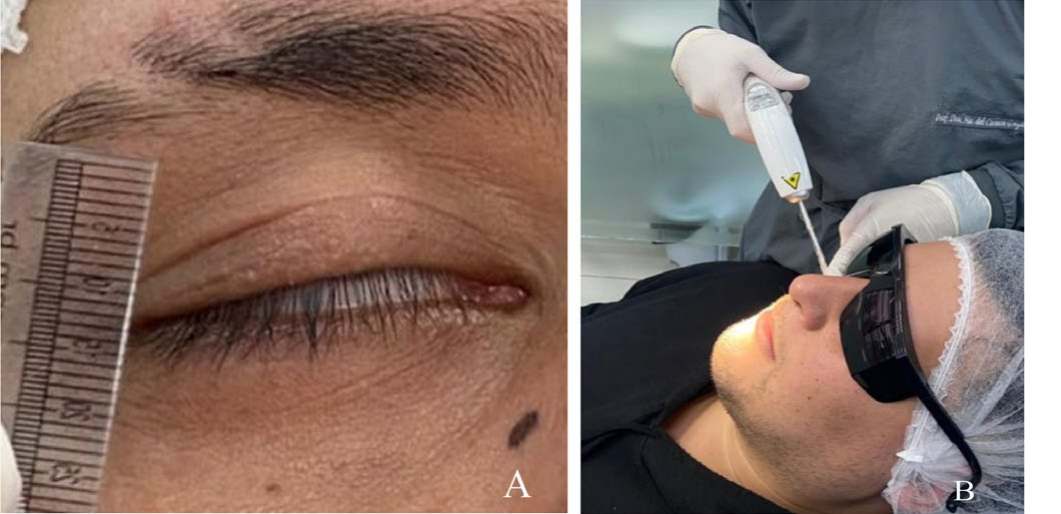
A) Medición del distanciamiento palpebral. B) Aplicación del láser en la curvatura malar.

El láser se configuró en la misma frecuencia para todas las sesiones: 4 julios para aplicación en 4 puntos siguiendo la curvatura del malar cada 1 cm. Antes de la aplicación del láser, se realizó la medición del distanciamiento palpebral, que fue de 3 mm (Figuras 2a y 2b)

**Figura 3:**
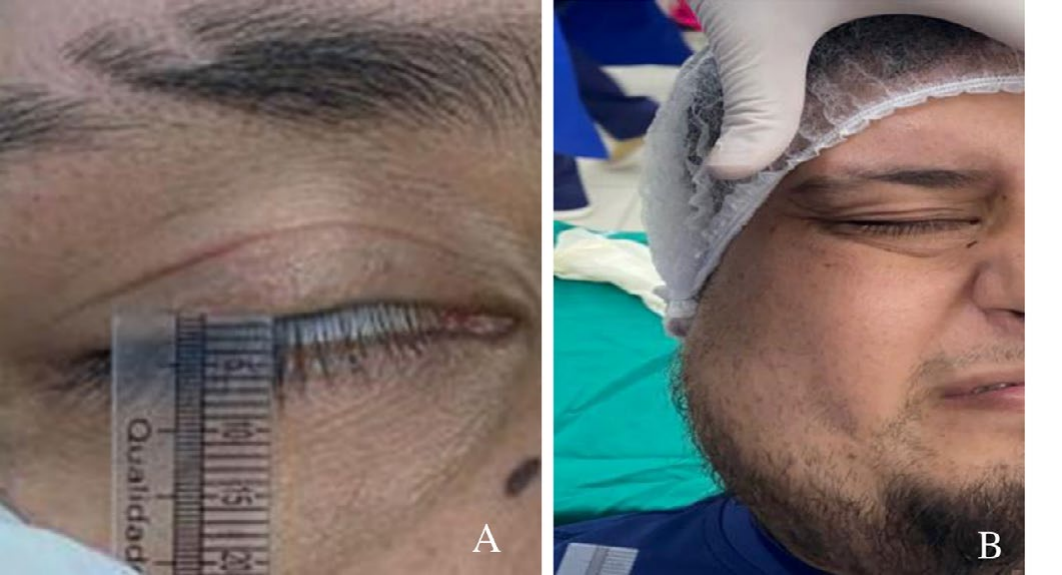
A) Distanciamiento palpebral de 2 mm. B) Cierre total palpebral con esfuerzo.

En la segunda sesión de la aplicación del láser ya se observó un mejor cierre, con un distanciamiento palpebral de unos 2 mm. En la tercera sesión aún presentó el distanciamiento mencionado, pero se logró el cierre palpebral con fuerza y culminó sus sesiones de fisioterapia de electroestimulación (Figuras 3a y 3b).

En la cuarta sesión se observó una distancia palpebral menor a 1,5 mm, mayor contracción del músculo orbicular y se indicaron nuevamente sesiones de fisioterapia de electroestimulación, ya que persistió el dolor en la zona de músculo esternocleidomastoideo a nivel de su inserción superior.

**Figura 4:**
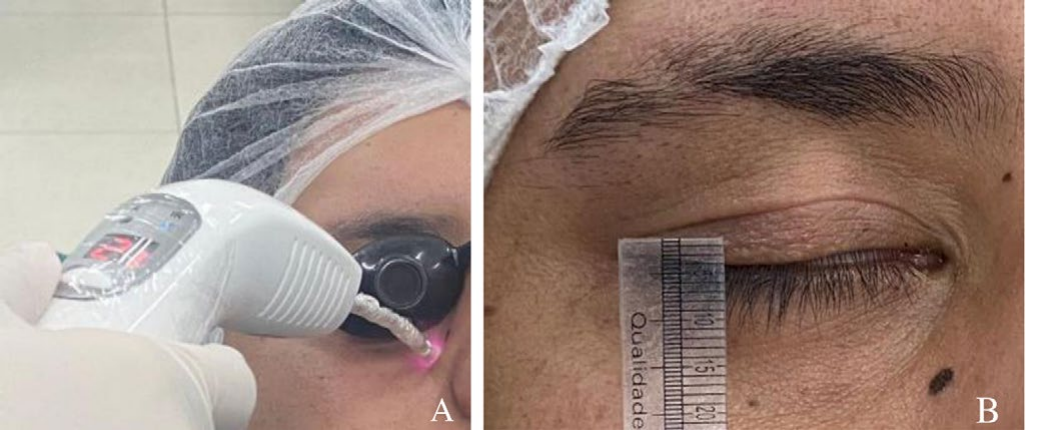
A) Aplicación de última sesión de láser. B) Cierre total palpebral en reposo.

En la quinta y última sesión se observó una distancia palpebral de 1 mm. El paciente refirió haber recuperado la sensibilidad alrededor de la zona palpebral, se corroboró que este pudo realizar el cierre con un ligero esfuerzo, pero aún no consiguió el cierre total del párpado en reposo; por ende, se sugirió el seguimiento clínico (Figuras 4a y 4b).

### Segundo caso

Una paciente de 16 años acudió a la clínica de patología bucal y refirió poca sensibilidad en el lado izquierdo de la región mentoniana, tras dos semanas de evolución debido a la extirpación de un tumor odontogénico en la zona de los molares de la hemiarcada izquierda. La paciente ya había sido diagnosticada con parestesia por su médico de cabecera. 

En el examen clínico extraoral se observó una ligera asimetría del lado izquierdo y, a la palpación, se distinguió una sobreelevación del tejido óseo en el cuerpo de la mandíbula. El examen clínico intraoral reveló la ausencia de las piezas dentarias 36 (primer molar inferior izquierdo) y 37 (segundo molar inferior izquierdo), extraídos en la intervención, una disminución del reborde alveolar en la zona y tejido cicatrizal en la mucosa de 3 cm, vestigio de la intervención mencionada. 

**Figura 5:**
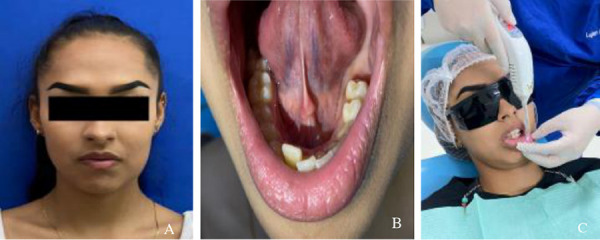
A) Fotografía extraoral donde se percibe un ligero abultamiento facial en el lado izquierdo. B) Fotografía intraoral donde se observa la ausencia de piezas dentarias y la disminución del reborde alveolar izquierdo. C) Aplicación de láser en las zonas afectadas.

Se procedió a realizar el tratamiento con láser de baja potencia en las siguientes zonas afectadas: la zona del trigémino, en 3 puntos, con una densidad de energía de 2 julios; el área del mentoniano, en 9 puntos, con una densidad de energía de 3 julios; y la mucosa del labio, en 4 puntos, con una densidad de energía de 2 julios y una distancia de 3 cm entre punto y punto. El tipo de contacto fue perpendicular y la emisión, continua (Figuras 5a, 5b y 5c).

**Figura 6:**
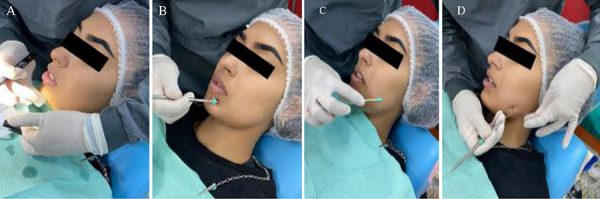
Pruebas térmica y mecánicas realizadas. A) Prueba con frío. B) Prueba con el mango del espejo. C) Prueba con un hisopo. D) Prueba con la punta de la sonda exploradora.

Se programaron diez sesiones de láser y, al final de la última sesión, se corroboró la mejoría de la zona afectada realizando cinco pruebas diferentes. Primero, se efectuó una prueba con frío; la segunda fue una prueba de presión con el mango del espejo, que abarcaba una mayor extensión; la tercera usó una prueba con un hisopo que se deslizó por la zona donde no sentía el estímulo; la cuarta fue una prueba de presión mucho más específica, con la punta de una sonda periodontal, para que la paciente mencionara las zonas donde tenía sensibilidad y las que no; y, por último, en la quinta, se presionó con las puntas de la pinza de algodón sobre la zona afectada para que la paciente indicara si podía diferenciar los dos puntos de presión al mismo tiempo (Figuras 6a, 6b, 6c y 6d).

Con la terapéutica, la evaluación y los datos proporcionados, la paciente refiere haber recuperado la sensibilidad en la zona en un 95%, con zonas muy pequeñas aún con la sensación de parestesia. 

Los pacientes aceptaron su participación firmando el consentimiento informado de la institución, en el cual se especifica que toda información proveída por ellos y las fotografías del caso podían ser utilizadas con fines educativos o de investigación. Asimismo, se cuenta con la aprobación (P-009-2024) del Comité de Ética en Investigación (CEI) de la Facultad de Odontología de la Universidad Nacional de Asunción.

## DISCUSIÓN

En el campo odontológico, puede ocurrir la pérdida de la función sensorial o motora de los nervios de la región facial, como resultado de ciertos procedimientos odontológicos. Esto puede generar alteraciones importantes para los pacientes, con diversos grados de disfunción ^8^.

Los trastornos neuropáticos que afectan con mayor frecuencia la práctica clínica de los cirujanos dentistas son la parestesia, la parálisis facial y la neuralgia ^9^^-^^11^. Estos trastornos neuropáticos representan un desafío para el cirujano dentista, ya que hay diversos tipos de tratamientos que pueden ser aplicados, según el caso ^12^. 

Las aplicaciones del láser de baja intensidad, en sus inicios, se centraron en los tejidos blandos; sin embargo, actualmente son de gran utilidad también para tejidos duros, como en complicaciones neurosensoriales ^13^. En el caso presentado, por ejemplo, la terapia láser ha ayudado a la recuperación de los pacientes. 

Algunos estudios reportan el beneficio de la aplicación de la terapia con láser en la recuperación neurosensorial de pacientes que fueron sometidos a cirugías bucales, como cirugía ortognática, en la que se observó mejoras significativas entre sesiones de la aplicación y la recuperación de la percepción neurosensorial ^14^^-^^16^. 

Las principales ventajas de la terapia con láser son la ausencia de contraindicaciones y efectos secundarios, ya que es un tratamiento totalmente no invasivo e indoloro para el paciente, y muestra, en la mayoría de los casos, mejoras significativas en corto tiempo tras su aplicación ^17^^,^^18^. Asimismo, se ha concluido que la aplicación de la terapia láser puede mejorar la percepción de los mecanorreceptores en aberraciones sensoriales de larga duración ^(19, 20)^. 

En el campo estomatológico es imprescindible la utilización de nuevas tecnologías como evidenciar científicamente los avances y beneficios que las mismas representan para ciertas patologías atípicas dentro de nuestro campo que podrían representar un retro en el tratamiento. El beneficio del láser de baja potencia es evidente ante este tipo de patologías como de otras por sus múltiples ventajas, es importante también recalcar el manejo multidisciplinario que se debe otorgar a este tipo de casos.

## CONCLUSIÓN

La aplicación del láser de baja potencia presenta resultados idóneos en diversas áreas del campo estomatológico. Al ser un tratamiento no invasivo, indoloro y con sesiones de corta duración, estimula al paciente a continuar con el tratamiento hasta estar casi totalmente rehabilitado. Si bien no es el único tratamiento que se podría utilizar para el tratamiento y manejo de neuropatías, ya que la evidencia también habla del manejo farmacológico, es un excelente coadyuvante para lograr la rehabilitación del paciente en menor tiempo.
